# Creating a Framework for Treating Autoimmune Gastritis—The Case for Replacing Lost Acid

**DOI:** 10.3390/nu16050662

**Published:** 2024-02-27

**Authors:** Lori Taylor, Andrew McCaddon, Bruce H. R. Wolffenbuttel

**Affiliations:** 1 Faculty of Integrative and Functional Nutrition, Saybrook University, Pasadena, CA 91103, USA; 2 Faculty of Social and Life Sciences, Wrexham University, Wrexham LL11 2AW, UK; mccaddon@sky.com; 3 University of Groningen, University Medical Center Groningen, 9713 GZ Groningen, The Netherlands

**Keywords:** autoimmune gastritis, pernicious anemia, atrophic gastritis, hypochlorhydria, betaine hydrochloride, iron deficiency, B12 deficiency, proton pump inhibitors, H2 receptor antagonists, vitamin C

## Abstract

Autoimmune gastritis (AIG) is characterized by the destruction of gastric parietal cells, resulting in hypochlorhydria and eventual achlorhydria, as oxyntic glands in the corpus are destroyed and become atrophic. The permanent loss of gastric acid has many impacts—both theoretical and documented. The most concerning of these are hypergastrinemia and increased N-nitroso compounds, both of which increase the risk of gastric cancers. While known deficiencies of B12 and iron are often replaced in AIG, acid is not. Moreover, patients with AIG are often prescribed acid suppression for a stomach that is decidedly no longer acidic, worsening the sequelae of gastric atrophy. Betaine hydrochloride (BHCL) is a short-acting acidifying agent, available over the counter in capsule form. Mealtime acid supplementation has an historic basis and could ameliorate many AIG-related gastrointestinal symptoms. Theoretically, acidification could also reduce the potential for hypergastrinemia and the production of N-nitroso compounds, consequently reducing the risk of gastric cancers. Supplemental vitamin C may also help in preventing gastric N-nitroso formation, regardless of the gastric pH. This narrative review describes the functions of gastric acid in gastrointestinal and immune health, documents the effects of hypochlorhydria in AIG, and proposes potential options for safely re-establishing the acid milieu of the stomach for patients with AIG.

## 1. Background

In normal gastric physiology, parietal cells located in the oxyntic (acid-secreting) glands of the corpus produce hydrochloric acid (HCL) and intrinsic factor (IF). The secretion of HCL by the parietal H+/K+ ATPase creates an acidic milieu that acts as a barrier to ingested bacteria, supports the digestion of proteins, and releases nutrients from their organic complexes, allowing for their absorption. Parietal cells also produce IF, a protein required for vitamin B12 absorption in the terminal ileum. Gastric pH is controlled primarily by the hormone gastrin, released by G cells in the antrum when the gastric pH > 4. Gastrin stimulates enterochromaffin-like (ECL) cells in the oxyntic glands to release histamine, which in turn signals adjacent parietal cells to secrete acid.

In AIG, anti-parietal cell antibodies (PCA) target the H+/K+ ATPase, leading to the destruction of parietal cells by autoreactive T cells, causing inflammation throughout the corpus and progressive atrophy of the oxyntic glands. Anti-intrinsic factor antibodies (IFA) may also develop, further diminishing available IF. Without sufficient acid, the stomach becomes chronically hypochlorhydric and, eventually, achlorhydric. Given its numerous roles, it is not surprising that the lack of HCL has a profound effect on the function of the gastrointestinal (GI) tract. In AIG, the lack of acid and IF cause iron, vitamin B12, and other micronutrient deficiencies [1], as well as a host of primarily upper GI symptoms [2]. The chronically elevated intragastric pH continues to stimulate the release of gastrin, resulting in hypergastrinemia which leads to ECL hyperplasia and increases the risk of developing type 1 neuroendocrine tumors (NETs) [3].

## 2. Presentation and Diagnosis of AIG

AIG can be difficult to diagnose, and is likely underdiagnosed [4]. The classic macrocytic anemia of pernicious anemia (PA) is a late-stage manifestation of AIG and only occurs in 15–25% of cases; a lack of macrocytosis or anemia does not rule out AIG [5]. Patients with AIG often present with diffuse constitutional and nonspecific upper GI symptoms, with refractory iron deficiency anemia being present in 15–27% of these patients and as many as 57% of them having iron deficiency without anemia [6]. Iron deficiency generally precedes B12 deficiency, especially in menstruating women. Low serum ferritin, low transferrin saturation, and microcytic anemia were present in nearly half of AIG patients studied by Hershko, et al. [7]. The anemia and iron deficiency of AIG are often revealed by, but misattributed to, menstrual blood loss, which is pertinent, as AIG is 2–3 times more common in women than in men [6]. It should be noted that AIG is much more commonly found in idiopathic iron deficiency than celiac disease [8]. B12 deficiency itself is challenging to detect, as it lacks a gold standard for diagnosis, with laboratory measures of B12 status being less reliable than the overall clinical picture [9]. Complicating matters further, the microcytosis of iron deficiency and the macrocytosis of B12 deficiency combined can produce a normocytic mean corpuscular volume in AIG [4].

The combined effect of iron and B12 deficiencies produces symptoms of fatigue, weakness, shortness of breath, hair loss, or sleep disorders [10]. Prolonged B12 deficiency can cause a host of neurologic symptoms, including changes in cognitive function and mood disorders, as well as sensory, motor, and autonomic neuropathies. These symptoms can be best alleviated with parenteral B12 treatment but can take months or years to completely resolve. If B12 deficiency is severe or longstanding, some neurological damage may be permanent [9].

No diagnostic standard has been established in AIG. As in B12 deficiency, the overall clinical picture is most important. Orgler et al. recommend that patients with nonspecific GI symptoms and/or iron deficiency—with or without anemia—should be evaluated for AIG through laboratory testing that includes PCA, IFA, and serum gastrin, in addition to a complete blood count, measures of iron status (transferrin saturation, transferrin, ferritin), and measures of B12 status [10]. Elevated PCA levels are generally indicative of AIG; however, sensitivity and specificity may differ considerably between assays [11]. Conversely, it should be noted that many individuals with a clinical diagnosis of PA have been found to be antibody-negative, especially those of Western European descent [12,13].

Half of patients with AIG do not report upper GI symptoms [2,14], making it difficult to identify which patients are appropriate for upper endoscopy. The American Gastroenterological Association (AGA) recommends that a diagnosis of atrophic gastritis be explored in patients with unexplained B12 or iron deficiencies [15]. Antico et al. developed a serologic gastric biopsy for AIG based on the heterogeneity of AIG disease presentation. They recommend that patients with risk factors for AIG and/or refractory B12 or iron deficiency anemia be tested for PCA, IFA, serum gastrin, and anti-*Helicobacter pylori* antibodies as a way to identify patients appropriate for endoscopy [16]. Upper GI endoscopy is warranted to obtain biopsies for the histologic confirmation of AIG and evaluation for NETs. In the early stages, however, AIG is difficult to distinguish endoscopically and histologically from healthy or mildly inflamed mucosa [6].

Lenti et al. provide an excellent guide to a differential diagnosis of AIG that includes clinical, serologic, endoscopic, and histopathologic characteristics of similar diseases, noting that *H. pylori* and AIG can co-exist [6]. The AGA guidelines recommend that all patients with atrophic gastritis be evaluated for *H. pylori* infection and treated for eradication if positive [15]. Orgler et al. are in the process of developing a symptom scoring instrument to provide a more systematic approach to AIG diagnosis [10].

Effective treatment of AIG requires parenteral replacement of B12 and, in many cases, parenteral iron, as hypochlorhydria and a lack of IF make both nutrients refractory to oral supplementation [6]. Conventional treatment however has not yet considered the replacement of lost acid, focusing mainly on B12 and iron.

## 3. Functions of Gastric Acid and Effects of Hypochlorhydria

While gastric acid is best known for its role in protein digestion, the acid milieu of the stomach is a necessary regulator of the overall digestive function, immune health, and the assimilation of micronutrients. As such, the consequences of hypochlorhydria are profound and extend beyond the GI tract itself. Table 1 describes the functions of normal gastric acidity and the consequences of hypochlorhydria from studies on patients taking proton pump inhibitors (PPIs) or those with oxyntic atrophy (due to *H. pylori* infection, AIG, or other causes).

### Effects of pH on Vitamin C and Fate of Gastric Nitrites

Vitamin C plays an impressive role in gastric health and its pH-dependent function in the stomach merits elaboration. In patients with a normal gastric histology, total vitamin C is concentrated in the stomach at three times the level in the plasma [32]. The presumed role of this concentration is to prevent the synthesis of N-nitroso compounds, neutralize exogenous reactive oxygen species, and prevent the oxidation of non-heme iron, making it bioavailable [33].

Nitrites are strong nitrosating agents, generated by the oral microbiota from dietary nitrates found in vegetables [34]. Vitamin C reacts with nitrites in the stomach, effectively preventing the creation of carcinogenic N-nitroso compounds, namely, nitrosamines [35]. Vitamin C has a pKa of 4.29 and, at normal gastric pH of 1–2, exists primarily as ascorbic acid. Under these conditions, gastric nitrites are reduced by ascorbic acid to form constitutive nitric oxide (NO), which is absorbed by the gastric mucosa [36]. NO stimulates gastric mucus secretion and is necessary for the reduction in intragastric pressure (IGP) which allows for gastric accommodation when food is ingested [19].

As food dilutes the gastric juice, the pH rises to 3–5, and the ascorbate anion becomes the predominant form of vitamin C. Ascorbate reacts rapidly with the available nitrite, at a rate 240 times faster than ascorbic acid, effectively scavenging all available nitrites in the stomach [37]. As long as ascorbate is present, it can prevent nitrosation, even at a neutral pH [38].

The conversion from ascorbic acid to ascorbate anion is pH-dependent and reversible, but at high pH, vitamin C is irreversibly oxidized to diketogulonic acid and thus unavailable as a reducing agent. Moreover, the increased pH results in the gastric overgrowth of nitrate-producing bacteria from the mouth [31]. While an acidic stomach preserves both the ability to produce constitutive NO and the ability to prevent the creation of nitrosamines, a hypochlorhydric stomach is impaired on both counts.

## 4. The Effects of Parietal Cell Loss in AIG

### 4.1. Near Achlorhydria

Hypochlorhydria is perhaps the earliest sign of AIG and may precede the development of B12 deficiency by several years [39]. Hypochlorhydria is typically defined as a gastric pH > 4, with achlorhydria defined as a pH > 7 [21]. Measuring the level of hypochlorhydria in patients with AIG is a recent development. Tenca et al. used multichannel intra-luminal impedance pH (MII-pH) monitoring to study 41 AIG patients with upper GI symptoms. These patients fasted for 12 h prior to testing, and those on anti-secretory therapy were fasted for an appropriate time for their drug type (10 days for PPIs, 2 days for H2 receptor antagonists). The researchers found the 24 h median intragastric pH in patients with AIG to be 6.3, nearly achlorhydric [40]. Kulnigg-Dabsch et al. followed on, measuring pH via a test strip in collected gastric aspirates. In their retrospective analysis of 373 premenopausal women with idiopathic iron deficiency (where 6.2% were found to have AIG), 26 patients underwent endoscopy, with 20 patients undergoing pH strip testing of the aspirates. Twelve patients were found to have histologically confirmed AIG. Of these, five had hypochlorhydria (here defined as a pH 3–5) and seven had achlorhydria [8].

### 4.2. Upper GI Symptoms

Upper GI symptoms are common in AIG. Dyspepsia occurs at a rate of approximately 20–30% in the general population [41], but much more frequently in AIG. In a cohort of 109 patients with AIG studied by Soykan et al., the most frequently reported symptoms were abdominal in nature, with nearly 47% of patients reporting abdominal bloating either alone or in combination with abdominal pain, nausea, or constipation [14]. In a cross-sectional study of 379 patients with AIG by Carabotti et al., more than half had one or more GI symptoms. Almost 70% of symptomatic patients had exclusively upper GI symptoms, of which 60% reported post-prandial dyspepsia (characterized by early satiety and post-prandial fullness). Almost 16% of patients reported only lower GI symptoms, and 15% reported both upper and lower GI symptoms. Those under the age of 55, of a female sex, with non-smoking status, and those with anemia were more likely to have dyspepsia [2]. In an earlier study of 99 patients with AIG by Miceli et al., GI complaints were also frequent, with 35% of patients reporting epigastric pain, 24% reporting heartburn, 22% reporting nausea, and 10% reporting early satiety [42].

Delayed gastric emptying is seen in both autoimmune and non-autoimmune atrophic gastritis, suggesting the loss of acid as a probable cause [2]. Kalkan et al. measured gastric emptying of a scrambled egg meal in patients with AIG, where patients with functional dyspepsia acted as controls. A total of 80% of the patients with AIG showed delayed gastric emptying of the scrambled egg meal, with a median gastric emptying time of over 2 h and over 60% retention of contents, compared to the control group with functional dyspepsia, which had a median emptying time of 81 min and 20% retention of contents. Delayed emptying was worse in AIG patients with more upper GI symptoms; the predictors of delayed emptying included higher gastrin levels [43]. Soykan et al. found retained food after a 12 h fast in three out of fifty-one AIG patients with abdominal symptoms, indicating the presence of gastroparesis [14].

Gastroesophageal reflux disease (GERD) commonly occurs in hypochlorhydria, consistently affecting about one quarter of patients with AIG. Twenty-four percent of AIG patients studied by Miceli et al. complained of heartburn [42]. Carabotti et al. found that 24% of patients with atrophic body gastritis (from AIG and/or *H. pylori*) had heartburn or regurgitation [26]. In the MII-pH monitoring study, Tenca et al. found ten out of forty-one patients (24%) to have GERD—nine with non-acidic reflux and one with slightly acidic reflux (pH < 4). Despite hypochlorhydria, 61% of the patients studied by Tenca et al. had been prescribed antacid therapy, predominantly PPIs. The authors stressed that PPIs are inappropriate in a stomach where acid is already suppressed. PPI therapy was stopped in all patients in the study except for one patient with acid reflux. Patients were followed for an average of 20 months after the cessation of PPI therapy and reported no change in their upper GI symptoms [40].

### 4.3. Immune Insult

#### 4.3.1. Gastric Dysbiosis

Research on the gastric microbiome in AIG is still in its early stages [5] but paints a picture of gastric bacterial overgrowth, nitrification, and increased oral and pathogenic bacteria. Stockbruegger et al. were among the earliest to identify overgrowth of nitrosating bacteria in the gastric and duodenal aspirates of patients with PA. Nitrite concentration and the mean count of nitrate-producing bacteria were significantly higher in patients with PA than in a control group of peptic ulcer patients [44]. Mitsui et al. studied the effect of hypochlorhydria on breath nitrous oxide concentrations in healthy patients, in those with partial gastrectomy, and in patients with gastric atrophy (where low pepsinogen levels were used as a proxy for atrophy and include gastritis caused by *H. pylori* as well as AIG). Patients with atrophy had significantly higher mean breath concentrations of nitrous oxide than the controls, though their levels were not as high as those of gastrectomy patients [45]. These findings pose a new question: -might the nitrous oxide created in atrophic stomachs be significant enough to impact B12 status, as nitrous oxide can oxidize the cobalt in B12 and irreversibly inactivate the methionine synthase enzyme [46]?

More recent studies have used 16S ribosomal RNA sequencing to look specifically at the taxa of species present in the stomachs of patients with AIG and have found a similar picture of overgrowth of both commensals and pathogens. Parsons et al. sequenced gastric corpus biopsies and found a heterogenous bacterial composition, with 266 genera identified; 57 of those genera were more frequently found in normal acidic stomachs. Bacterial diversity was similar in patients with AIG and controls; however, those with AIG showed a greater abundance of bacteria than the controls. The patients with AIG also had proportionally more *Streptococcus* species than any other patient group [47]. This is notable as *Streptococci* are prominent in the oral microbiota and would normally be killed in an acidic stomach [5]. AIG patients were the only group in which bacterial taxa were lost [47]. In a retrospective cohort study of patients undergoing endoscopy either for gastroesophageal symptoms or cancer screening, Tsuboi et al. sequenced the gastric biopsies of patients with corpus predominant (“open-type”) atrophy, which occurs in both AIG and *H. pylori*. Sixty-two percent of the patients with AIG had a previous or concurrent *H. pylori* infection. Researchers found higher proportions of four genera in the patients with AIG, with three of those genera appearing only in patients with AIG. *Streptococcus*, again, was predominant, as was *Bacillus* [48]. Arai et al. studied 261 patients with gastric cancer and found a greater abundance of *Bacillus cereus* in the 8 patients with cancer and pure AIG (no *H. pylori* history) [49]. The findings of *Bacillus cereus* are concerning as it is a pathogen that can cause a spectrum of disease, from food-borne illness to sepsis [50].

#### 4.3.2. Cancer

AIG is considered a precancerous condition due to atrophy, hypochlorhydria, chronic inflammation, and ECL hyperplasia, all of which increase the risk of type 1 neuroendocrine tumors (NETs), which are generally benign but require monitoring [5]. From 4 to 12% of patients with AIG have type 1 NETs [6]. The increase in nitrosation may also increase the risk of gastric adenocarcinoma, though the Correa model of gastric carcinogenesis refers to multifocal (corpus + antral) atrophy as a requirement, while in AIG, atrophy is confined to the corpus. As such, the increased risk of gastric adenocarcinoma is under debate [39,51]. The risk of developing type 1 NETs in AIG is from 0.4–7%, and the incidence of gastric adenocarcinoma ranges from 0 to 1.8% per year [5].

Due to the increased risk of gastric cancers, endoscopy is recommended for patients with an AIG diagnosis. European guidelines for patients with AIG recommend stratification for gastric cancer risk, and upper endoscopy with biopsies as per the updated Sydney system [52] every 3–5 years [53]. The American Gastroenterological Association (AGA) proposes similar surveillance for advanced atrophic gastritis, with an upper endoscopy every 3 years. For patients with either a PA or an AIG diagnosis, AGA recommends baseline endoscopy with a biopsy to assess for atrophy and the presence of NETs, with a 1–2 year endoscopy interval recommended for those with NETs [15].

### 4.4. Micronutrient Deficiencies

It is well known that the hypochlorhydria of AIG contributes to B12 deficiency, but it is perhaps less known that hypochlorhydria first causes iron deficiency. Approximately 50% of patients with AIG are iron deficient, and 37–60% are B12 deficient [39]. Given that both are hematopoietic nutrients, it is surprising that anemia is found in only half of AIG patients at diagnosis [6].

Iron deficiency without anemia is common in AIG. Kulnigg-Dabsch et al. studied 373 patients with iron deficiency (defined as serum ferritin < 50 µg/L and transferrin saturation < 15%) who were positive for parietal cell antibodies (PCA). Of these patients, 95% were female, and the cohort had a normal mean B12 level of 400 pg/mL. All the patients were iron deficient as per the inclusion criteria, but only half had anemia. A surprise finding of the study was that PCA served as an accurate screening tool for AIG in patients with iron deficiency, especially when the PCA was extremely elevated at >100 units/mL [8]. This study illustrates the challenge in diagnosing AIG before it leads to PA, given that nearly all the subjects had a normal B12 status and only half had anemia.

Cavalcoli et al. studied micronutrient deficiencies in AIG and found that, in addition to B12 and iron, deficiencies in vitamin C, calcium, and vitamin D may also be present [54]. Vitamin C, as previously described, is profoundly affected by hypochlorhydria and may become irreversibly oxidized in an AIG stomach. The solubility of calcium (and other minerals) is likely affected by hypochlorhydria. Further, vitamin D3 levels are lower in AIG than in any other type of gastritis, with levels half that of normal controls [55]. It is unclear whether vitamin D3 deficiency is a cause of AIG, an effect, or both. As vitamin D3 is necessary for calcium absorption, deficiency in vitamin D3 could also account for diminished calcium status [54]. To further complicate matters, calcium supplementation is not straightforward in hypochlorhydria. Calcium supplements are antacid in nature and taking calcium at meals may further alkalinize the stomach, assuming that calcium supplements can dissolve without sufficient acid. Calcium carbonate taken on an empty stomach is not absorbed well in hypochlorhydria, though more acidic calcium citrate forms are more effective [56].

Vitamin C supplementation may be helpful in AIG as this vitamin is inactivated in a hypochlorhydric stomach, and vitamin C inhibits N-nitroso formation that is more prevalent in hypochlorhydria [38]. Gastritis itself is associated with lower concentrations of vitamin C in the gastric juice (but not in the plasma) and may be related to inflammation, utilization by bacteria, hypochlorhydria, or a combination [33]. Waring et al. analyzed the vitamin C in the serum, gastric mucosa, and gastric juice of 48 patients with and without gastritis, where 75% of the patients with gastritis had *H. pylori* gastritis. While patients were not sorted by disease, they were sorted by their gastric pH. The patients with gastritis and hypochlorhydria (pH > 4) had very low levels of total vitamin C (ascorbic acid and dehydroascorbate) in their gastric juice, with almost no ascorbic acid, and thus, no protection against nitrosation. A later, different set of subjects with and without gastritis were given 500 mg of ascorbic acid twice daily, and their vitamin C levels were assessed after two weeks. Supplementation increased the total vitamin C and ascorbic acid in the plasma and gastric juice of both groups, though the gastric juice concentration of vitamin C in the patients with gastritis was lower than in those without gastritis. No patients in the supplementation study were hypochlorhydric, so it is not confirmed that gastric vitamin C concentrations can be increased by supplementation in hypochlorhydria [57]. Still, the authors of the study support vitamin C supplementation to decrease N-nitroso formation for cancer prevention and posit that clearing *H. pylori* would likely allow vitamin C to rise to greater levels in the gastric juice. A 1000 mg/day intake of vitamin C is greater than the recommended daily allowance (RDA) of 75–90 mg/day but less than the upper limit intake (UL) of 2000 mg/day [58] and poses little risk or cost in comparison to its potential benefit.

### 4.5. The AIG Stomach

The AIG stomach is atrophic, hypochlorhydric, and dysbiotic, causing both impaired digestion and motility. The loss of gastric acid alters GI tract signaling and GI hormone activation, with the potential to impair downstream intestinal, pancreatic, and biliary responses. The lack of stomach acid increases the risk of deficiencies in vitamin C, iron, and other micronutrients, both by their incomplete digestion from food complexes and by their reduced bioavailability due to oxidation. The atrophied AIG stomach is considered preneoplastic due to hypergastrinemia, ECL hyperplasia, and the concomitant decrease in vitamin C and increased production of N-nitroso compounds.

The secretion of stomach acid has the greatest influence on diseases outside the stomach compared to all other gastric functions [19]. Given that the loss of gastric acid is a common mechanism for nearly all the sequelae of AIG, replacing gastric acid should be considered for its potential to ameliorate these effects.

## 5. Replace, Don’t Reduce, Mealtime Gastric Acid

### 5.1. Avoid PPIs

The use of PPIs is prevalent in medicine and often recommended without an evidence-based indication [17]. Additionally, PPIs are now available without prescription in the US. It is not surprising then that as many as 61% of AIG patients use PPIs, despite the fact that acid suppression would worsen hypochlorhydria and that most GERD in AIG is non-acidic unless proven otherwise during endoscopy [40]. Treatment of AIG with PPIs is not indicated, and limited observational reports find it ineffective for relieving dyspepsia; additionally, it may worsen hypergastrinemia and increase the risk of gastric cancer [59,60].

PPIs are indicated in the treatment of ulcers, which are not caused by AIG. Before beginning mealtime acidification, it is prudent to ensure that the patient with AIG does not also have ulcers, which are predominantly caused by *H. pylori* and/or the use of nonsteroidal anti-inflammatory drugs (NSAIDs) [61]. Ulcers would be apparent with the baseline upper endoscopy recommended for patients diagnosed with AIG and should be treated prior to any mealtime acidification.

### 5.2. Historic Use of Gastric Acidification with HCL

Hydrochloric acid replacement therapy dates back to the late 19th century, when it was administered to improve digestion, release pepsin, normalize motility, and act as a gastric antiseptic. Dilute solutions of 10% HCL in water were given before meals, three times daily, to tolerance, with one cup of 10% HCL having a pH ranging from 1.3 to 3.2. In 1918, Crohn found that diluted HCL supplementation did not improve protein digestion in patients with achlorhydria; this may be because the HCL solution is rapidly neutralized by the stomach [35]. In 1942, Westfall studied 300 patients with achlorhydria; 76% of them reported that their symptoms improved with HCL supplementation [39]. HCL supplementation was not able to improve non-heme iron absorption in patients with atrophic gastritis studied in the 1960s and 1970s [18]. The value of these studies for current-time medical treatments is quite limited, due to major changes in dietary habits over the last 50–100 years. Additionally, placebo-controlled studies were lacking in early research. It should also be noted that liquid solutions of HCL do not protect the teeth or esophagus, though cola beverages have a similar pH, around 2.5 [62].

### 5.3. The Evidence for Acidification with Betaine Hydrochloride

Betaine hydrochloride (BHCL) is the hydrochloride salt of the amino acid betaine, which dissociates to betaine and HCL in the stomach, quickly releasing H+. BHCL is available over the counter in capsule form and is often used as an acidifying treatment for dyspepsia. (Note that betaine HCL differs from the betaine anhydrous form used to safely treat homocystinuria, at dosages of 6–20 g/day [63].) BHCL remains widely available despite a 1993 ban on BHCL, dilute HCL solutions, pepsin, and other acidifiers by the US Food and Drug Administration, stating a lack of evidence for their status as “generally recognized as safe” (GRAS) [64]. BHCL is available for purchase online in capsules varying from 500 to 750 mg, with or without pepsin, with 1500 mg as the typical per-meal dosage recommended by manufacturers. Encapsulated BHCL has the advantage of protecting the teeth and the esophagus from the effects of HCL while allowing for a higher effective dose of acid: one 750 mg capsule can deliver nearly five times more acid to the stomach than acidic beverages [63].

Human studies of BHCL have been recently conducted to determine its efficacy as an acidifier to improve drug absorption in patients also taking PPIs. Drug studies typically assume an acidic gastric pH, and the common use of PPIs can both raise the pH > 4 and diminish drug absorption. In a pilot study, Yago et al. tested the effect of BHCL on six volunteers with normal gastric acidity reversed to a pH > 4 by the PPI rabeprazole. A 1500 mg dose of BHCL with pepsin was given to the participants on an empty stomach. Their gastric pH was then continuously monitored over a 2 h period by a Heidelberg capsule. BHCL was effective in acidifying the stomach—it lowered the gastric pH from 5.2 to 0.6 in a period of 30 min and reduced the pH to <3 within 6 min. The acidifying effect was transitory, with the gastric pH remaining under a value of 3 for 73 min and under 4 for 77. No significant adverse effects or GI side effects were observed [63]. In a follow-on randomized three-way crossover study, Yago et al. studied the effect of rabeprazole induced achlorhydria on the absorption of the leukemia drug dasatanib; 1500 mg of BHCL completely reversed the impact rabeprazole had on its absorption [65].

A further study by Faber et al. on the effect of BHCL and PPIs on absorption of antiretroviral medications found that a small meal blunted the acidifying effects of BHCL, and drug absorption was unable to overcome the buffering effect of the meal [66]. BHCL was given 10 min prior to the meal, so timing may have been an issue. Surofchy et al., from the same group, studied how quickly gastric pH returned to baseline after a 310-calorie breakfast meal, similar in composition to the meal from the Faber study. In a randomized, four-way crossover trial, nine subjects were given no PPIs or other medications but were given 1500, 3000, or 4500 mg doses of BHCL 15 min after a meal, with the controls receiving no BHCL. The control group returned to their baseline pH in 50 min. The BHCL dosages of 1500 and 3000 mg did not significantly reduce the time to return to the baseline. Only the 4500 mg dose of BHCL (six capsules) was able to return the pH to its pre-meal baseline faster, in 17 min. The subjects experienced no adverse effects from either the Heidelberg capsule or the BHCL, despite the high dosages [67]. Surofchy’s study suggests that BHCL does not improve acidification in a healthy stomach except in high amounts and that BHCL is well tolerated even in high amounts. The timing of BHCL appears to be important as well. The administration of BHCL 15 min after a meal may have lessened its effects in this study. Gastric HCL is typically secreted just prior to a meal and as a meal is ingested and is dispersed by contractions in the antrum. BHCL well-before or after a meal may not be properly dispersed.

Forssman et al. studied the effect of a 250 mg amino acid hydrochloride and 250 mg bovine protease supplement in patients with functional dyspepsia meeting the Rome III criteria. In this observational, noninterventional, post-marketing surveillance study, patients completed a validated gastrointestinal symptom score (GIS) instrument before treatment and after 3 weeks and 6 weeks of supplementation. There was no placebo group. Patients took the supplement three times daily, with the number of tablets taken at each meal determined by the patient’s physician and in accordance with usage recommendations, where the daily dosage could vary between three tablets (750 mg amino acid HCL) and twenty tablets (5000 mg amino acid HCL). A greater than 50% reduction in the GIS scores was found in 31% of the patients after 6 weeks and reached statistical significance. A total of 27 out of the 97 patients discontinued supplementation due to GI symptoms, which they attributed to either insufficient treatment or a worsening of symptoms, with treatment tolerability deemed good to moderate [68]. These results are difficult to interpret due to the wide range of dosages given and the lack of controls.

Despite the limited scope of BHCL studies in humans, BHCL appears to be tolerated in healthy stomachs and by the majority of subjects with functional dyspepsia. Experts in AIG recommend trials of BHCL to ameliorate the GI side effects of hypochlorhydria [10,39].

### 5.4. How to Use Betaine Hydrochloride

Guilliams, in his review of the subject, suggests that BHCL be taken immediately before a meal or divided and interspersed with a meal, which would be consistent with the progressive nature of acid secretion throughout a meal. Dosage is commonly determined through tolerance of an HCL challenge, where patients begin with one capsule of BHCL at a meal and monitor for a burning sensation in the stomach. If burning is not felt, patients then progressively add one capsule at each following meal until they feel a warming effect, to a maximum of 3000 mg of BHCL per meal [58].

An HCL challenge may not be best for patients with AIG, as it is not known if patients with AIG have normal sensitivity to warming or burning sensations. A more practical outcome-based strategy could involve titrating BHCL dosage to the size of the meal, using the fewest number of capsules needed to reduce bloating and upper GI symptoms. A “start low and go slow” approach seems reasonable, with an initial trial dose of 500–750 mg of BHCL per meal, titrating up to Guilliams’ maximum of 3000 mg per meal, with BHCL never being the first or last part of a meal but interspersed.

BHCL is most commonly available with pork-derived pepsin. BHCL without pepsin is harder to find but may be a better first choice to assess tolerance, as some patients may not tolerate the chronic use of pepsin, an active protease. Others may not want to consume a product derived from animals. The pepsin precursor pepsinogen is made by chief cells in the oxyntic glands, with pepsinogen I (PG I) made in the corpus and pepsinogen II (PG II) made in the pylorus. The serum levels of PG I are lowered in AIG, while the PG II levels are normal, consistent with corpus-restricted atrophy [69]. Decreased ratios of PG I–PG II can also be used for pre-endoscopic screening in AIG [70]. Exogenous pepsin therefore may not be needed, as the AIG stomach continues to make PG II for conversion by BHCL. Pepsin is inactivated at an alkaline pH [71], and if amenable to the patient, the inclusion of pepsin with BHCL should not be a barrier to empiric treatment.

### 5.5. Considerations for the Use of Betaine Hydrochloride in AIG

It is not known how BHCL is tolerated in AIG. Nearly one quarter of the patients with functional dyspepsia in the Forssman et al. study did not tolerate acidification [68]; AIG patients may have similar experiences. AIG is characterized by inflammation in the corpus, which is likely due to autoimmune attack but may also be due to the release of excess histamine by ECL cells. In a normal stomach, gastrin binds to ECL cells, causing them to release histamine, which then binds to parietal cells, causing them to secrete HCL. In gastric atrophy, however, there are fewer parietal cells receiving the histamine signal and more ECL cells producing histamine due to hypergastrinemia and/or ECL hyperplasia. This could result in an excess of inflammatory histamine, with no functional effect on acid secretion. In humans with atrophic gastritis, ECL hyperplasia has been associated with an increase in the amount of histamine produced in the fundus of the stomach and increased activity of histidine decarboxylase (HDC), the enzyme which synthesizes histamine. The number of mast cells in patients with ECL hyperplasia was unchanged, implying that the increased histamine was being synthesized within ECL cells [72]. In rats, HDC enzyme activity is increased by gastrin and remains increased for as long as gastrin is being secreted, with the longest time studied being 20 weeks [73].

Patients with AIG who do not tolerate BHCL (and perhaps all patients with AIG) may benefit from commonly used antihistamine H2 receptor antagonists (H2RAs) like famotidine. While H2RAs are anti-secretory, they can and perhaps should be empirically used alongside BHCL to dampen the inflammatory effect of excess histamine in hypergastrinemia and/or ECL hyperplasia. H2RAs have a short half-life of 1–2 h, and H2RAs’ antacid effects diminish within 7–14 days of treatment [74]. Taken at bedtime, H2RAs may ameliorate AIG inflammation while having a limited effect on the next day’s meals, and any effect on what acid secretion remains in AIG could also be ameliorated by BHCL. Sucralfate may be helpful in the short-term to diminish stomach inflammation but is not recommended for long-term use in AIG as it adsorbs and reduces the concentration of pepsin [75]. While the use of H2RAs in AIG has not yet been assessed in controlled trials, their use as an anti-inflammatory in AIG merits consideration on theoretical grounds.

Mealtime acidification with BHCL has the potential to reduce gastrin levels, control bacterial overgrowth in the stomach, and prevent the formation of N-nitroso compounds—all of which could reduce the risk of gastric cancer in AIG. The addition of supplemental vitamin C could additionally reduce cancer risk by impairing N-nitroso compound formation regardless of the gastric pH. BHCL taken with meals may also improve overall digestion, GI motility, and absorption of micronutrients. Clinical trials of BHCL in patients with AIG are needed to assess its efficacy. The empiric use of BHCL in patients with AIG is worthy of consideration as there is initial evidence that BHCL is well tolerated in humans and may ameliorate GI symptoms related to hypochlorhydria.

## 6. Conclusions

Autoimmune gastritis is not a gastritis that needs acid suppression; rather, it may benefit from acid supplementation. The loss of stomach acid in AIG precedes nutrient deficiencies by several years. Hypochlorhydria affects digestion, micronutrient absorption, risk factors for benign and perhaps malignant diseases, as well as a range of gastrointestinal complications.

Betaine hydrochloride is a simple and readily available encapsulated form of acidification. Studies in humans are limited but show that betaine hydrochloride appears to be generally well tolerated. This paper proposes empiric mealtime acidification with betaine hydrochloride for patients with autoimmune gastritis. Acidification may ameliorate GI dysfunction, improve nutrient deficiencies, and bolster immunity. Research is needed in patients with AIG to assess tolerance and efficacy. Supplemental vitamin C is worthy of consideration in AIG, as it may decrease cancer risk in hypochlorhydria and improve iron absorption.

Autoimmune gastritis is more than B12 and iron deficiency. It is also an acid deficiency with broad effects. It is important for patients with AIG that the scope of their symptoms is recognized and, where necessary, further investigated. Research is needed to elucidate the pathophysiology of hypochlorhydria in AIG, explore patient experience, and investigate therapeutic options.

## Figures and Tables

**Table 1:** Function of gastric acid and effects of hypochlorhydria.

Function of Gastric Acid	Normal Gastric Function	Hypochlorhydria (pH > 4)
***Immune functions***		
Microbial barrier Gastric pH 1–2	Kill ingested bacteria at a pH < 4 within 15 min [17] Reduce the amount of nitrosating bacteria coming from the oral cavity	Gastric, oral, and intestinal bacterial overgrowth [18,19] Increase in nitrosating bacteria; increased risk of gastric cancer [20]
Inhibits nitrosamine formation and supports the production of constitutive nitric oxide	Acidic pH favors the conversion of oral nitrites to nitric oxide, limiting the formation of nitrosated compounds NO is needed to reduce intragastric pressure for gastric accommodation of food; stimulates mucus secretion	Increased N-nitroso compound formation; increased risk of gastric cancer [20] Poor gastric accommodation, dysmotility, early satiety, gastroparesis [19]
Regulates the secretion of gastrin	Gastrin is not released at a pH < 4. Food intake increases gastric pH, causing the release of gastrin until the pH returns to <4 [21]	Hypergastrinemia, leading to the hyperplasia of adjacent enterochromaffin-like (ECL) cells. Increased risk of intestinal metaplasia and neuroendocrine tumors [19]
***Digestive functions***		
Denaturation of proteins Activation of pepsin from pepsinogen	Breaks down 3° and 4° structure of proteins [22] Pepsin digests proteins at an acidic pH 1.5–2; inactive at a pH > 6 [23]	Incomplete breakdown of protein structure ^†^ Incomplete digestion of proteins ^†^ Delayed gastric emptying [18], dyspepsia [24,25], gastro-esophageal reflux disease (GERD) [26]
Stimulates the activation and release of secretin	Secretin stimulates the pancreas to secrete water and HCO_3_^−^, creating a neutral pH for the small intestine(SI) [27]	Increased duodenal pH [18]; altered osmoregulation ^†^
***Micronutrient-related functions***		
Digestion of micronutrients	Remove B12, Fe from organic complexes	B12 [28], Fe [29], and other micronutrient deficiencies
Maintains a reduced state for the bioavailability of micronutrients	Non-heme Fe^3+^ reduced to soluble Fe^2+^ by acid and ascorbate anion. Acidic pH allows the formation of an Fe^3+^ chelate with ascorbic acid that is stable at the alkaline pH of the SI [29] Vitamin C is kept primarily in an ascorbic acid form or as ascorbate as the pH rises with food intake.	Non-heme Fe^3+^ precipitates at a pH > 3; only Fe^2+^ or chelated Fe^3+^ can be absorbed [29]. Iron deficiency that is often refractory to oral therapy [30] Alkaline gastric juice oxidizes vitamin C making it unavailable as an antioxidant. Ascorbate unavailable to bind to Fe^2+^ for stabilization at a pH > 3 and subsequent absorption; also unavailable to prevent N-nitroso formation or act as an antioxidant [31]

^†^ Theoretical.
